# Assessing the Effectiveness of Voice Therapy Techniques in Treating Dysphonia: An Otolaryngological Review

**DOI:** 10.7759/cureus.62041

**Published:** 2024-06-10

**Authors:** Omair H Al-Hussain

**Affiliations:** 1 Otolaryngology - Head and Neck Surgery, Faculty of Medicine, Imam Mohammad Ibn Saud Islamic University, Riyadh, SAU

**Keywords:** ent, beneficial, treatment, voice therapy, dysphonia

## Abstract

Dysphonia is a prevalent condition that can impact individuals across all age groups. It occurs when normal voice quality is altered, caused by structural and/or functional issues. Evaluation and assessment from clinicians are warranted if dysphonia persists for more than four weeks and/or is coupled with risk factors or other concerning clinical manifestations. Additionally, voice disorders can increase the risk of depression and anxiety disorders, as well as raise stress levels and lower self-reported health indicators. Dysphonia can have a substantial influence on interpersonal interactions and lower overall quality of life since effective communication relies significantly on spoken language. Hence, managing dysphonia is essential for enhancing communication abilities, improving quality of life, maintaining vocational functioning, promoting psychological well-being, and addressing underlying health concerns. Speech and language therapy, medical management, surgery, or a combination of the aforementioned are all possible treatments for dysphonia. Speech and language therapy is often the first-line treatment option for dysphonia patients who do not meet the criteria for surgical intervention. Voice therapy is often beneficial and remains the first line of treatment, even when patients approach with benign vocal fold nodules. A well-designed voice therapy program improves both the quality of life and vocal performance. The majority of the studies in the existing literature advocate for and report beneficial outcomes associated with voice therapy; however, more research is needed to provide evidence-based findings to guide clinical practice and achieve optimal outcomes. This comprehensive review elaborately highlights the utilization and efficacy of various voice therapeutic modalities utilized for the management of dysphonia in light of current literature.

## Introduction and background

Dysphonia, which includes the cardinal symptom of hoarseness, affects around 1% of all patients and has a lifetime incidence of approximately 30% [1]. It refers to any voice impairment, including hoarseness, restriction of vocal performance, or strained vocalization [1]. Hoarseness can stem from a range of factors, such as acute and chronic laryngitis, laryngopharyngeal reflux, functional dysphonia caused by vocal strain or misuse, vocal cord paralysis, and various laryngeal disorders and growths [2]. Hoarseness resulting from acute and chronic laryngitis accounts for 42% and 10% of instances, respectively, while for functional dysphonia the incidence is 30% [3]. Additionally, the percentages attributed to hoarseness caused by age-related physiological changes in the voice and psychogenic factors are 2% and 2-2.2%, respectively, benign (15%) and malignant tumors (3%), while vocal cord paresis is responsible for 5% of cases [1].

Voice disorders can lead to heightened susceptibility to depression and anxiety disorders, increased levels of stress, and diminished self-reported indicators of health. Effective communication heavily relies on spoken language, making dysphonia a significant challenge that can profoundly impact interpersonal interactions and diminish the overall quality of life [4]. Dysphonia, a condition affecting vocalization, manifests in various acoustic forms. Perello introduced a well-known classification of dysphonia into two categories: organic and functional. Later on, Majdevac proposed a classification based on the primary etiological factors [5]. Under Majdevac’s classification, dysphonias caused by primary functional disorders include hyperkinetic dysphonia grades I and II, hypokinetic dysphonia, contact hyperplastic dysphonia, and dysodic dysphonia, while dysphonias resulting from primary neurogenic disorders encompass central dysphonias spasmodic/spastic dysphonia, dysphonia related to myasthenia gravis, dysphonia within skull base syndromes, dysphonia due to unilateral/bilateral palsy of the inferior laryngeal nerve and dysphonia due to palsy of the superior laryngeal nerve. Primary psychogenic disorders lead to psychogenic aphonia, psychogenic dysphonia, and false mutation. Dysphonias caused by primary somatic disorders include those resulting from vocal cord insufficiency, vocal cord edema, laryngitis secondary functional, cord-ventricular voice, posttraumatic dysphonia, arthrogenic dysphonia, and presbyphonia [5].

Presentation of hoarseness is frequent in ENT clinics, with estimates suggesting that over 50,000 patients seek care for dysphonia annually at otolaryngology/voice clinics [6]. Adequate vocal function is deemed necessary for approximately one-third of the workforce to effectively carry out their job responsibilities. Evaluating and treating patients with hoarseness can be intricate and prolonged due to its often multifactorial etiology [6]. Managing or treating dysphonia is essential for enhancing communication abilities, improving quality of life, maintaining vocational functioning, promoting psychological well-being, and addressing underlying health concerns.

Treatment initiation should prioritize voice rest, with particular emphasis on avoiding whispering, coupled with interventions aimed at addressing potential underlying causes. For instance, hoarseness attributed to reflux may warrant the use of proton pump inhibitors, while vocal abuse-related issues benefit from adhering to proper vocal hygiene practices. However, in cases lacking clear indications, empirical use of antibiotics, oral corticosteroids, or proton pump inhibitors for treating hoarseness is not recommended. Visualization of the larynx and vocal folds should be conducted within three months if an etiology remains unidentified or if conservative measures prove ineffective. Patients experiencing symptoms persisting beyond two weeks, especially those with risk factors for dysplasia like tobacco or heavy alcohol use, may necessitate an earlier laryngoscopic assessment. Voice therapy represents an effective means of enhancing voice quality for individuals with dysphonia when conservative approaches prove inadequate, and it may serve as a preventive measure for high-risk groups such as vocalists or public speakers. Surgical intervention becomes necessary for addressing laryngeal or vocal fold dysplasia or malignancy, airway obstruction, or benign pathology [7].

Moreover, voice therapy has been proven to demonstrate effectiveness in enhancing both self-assessed and externally evaluated aspects of voice quality. Typically, after ruling out conditions necessitating surgical intervention, patients are commonly directed to a speech and language therapist for voice therapy [8]. Voice therapy treatments may be divided into three distinct categories: hygienic, which refers to modifying behaviors that can cause vocal fold injury; symptomatic, which includes treating faulty voice quality in the ensuing phonated voice; and physiologic, which is optimizing voice output [9]. A well-designed voice therapy program improves both the quality of life and vocal performance. Short-term voice therapy (less than three weeks) may be as effective as long-term programs, and telepractice voice therapy may be a feasible option to improve therapy adherence [10]. Present clinical practice guidelines also advocate that clinicians should recommend voice therapy for patients experiencing dysphonia stemming from conditions suitable for voice therapy intervention [11]. Voice therapy is a recommended treatment for dysphonia, which remains a prevalent condition encountered in clinical practice at otolaryngological clinics. Therefore, assessing the efficacy of this modality is vital. Hence, we aim to conduct this review to comprehensively analyze the existing literature to define the effectiveness of various voice therapeutic techniques utilized for the management of dysphonia and provide evidence-based findings that can guide clinical practice, achieve optimal outcomes for patients, and significantly improve their quality of life.

## Review

Almost 30% of the adult population will experience voice issues at some point in their lives, whether chronic (21.5%) or acute (78.5%). Their voices will not perform or sound as usual, which may influence their communication, work, and overall quality of life. Voice issues can have a significant detrimental influence on a person’s social interactions, emotional state, and health, analogous to other chronic conditions such as heart failure, angina, and chronic obstructive pulmonary disease. Behavioral voice therapy, directed by a speech-language pathologist, is frequently advised as the primary strategy for treating voice abnormalities, and when not, it is indicated in conjunction with medical or surgical treatment [12].

As it becomes more extensively understood that medical treatments should be reviewed scientifically, paramedical therapies must also be objectively evaluated following current evidence-based medicine standards. The evaluation of voice therapy falls under this rising focus. However, there have been relatively few studies on the effects of voice therapy [13]. Van Stan et al. have developed a taxonomy of voice therapy that organizes direct and indirect treatment into more specific components. Direct intervention is classified into five categories: auditory, somatosensory, musculoskeletal, respiratory, and vocal function. The indirect intervention comprises pedagogy and counseling elements. This taxonomy empowers clinicians and researchers to contemplate therapeutic options based on physiological goals [14].

In the past decade, two systematic studies of large patient groups have provided promising evidence for the efficacy of voice therapy. Ruotsalainen et al. conducted a systematic literature review and concluded that an amalgamation of direct and indirect voice treatment is the best available solution for functional dysphonia when compared to no intervention. However, these findings are based on three studies and only on self-assessment measures: the Vocal Performance Questionnaire and the Voice-Related Quality of Life [15]. Speyer conducted a review of functional and organic dysphonia and discovered that direct voice therapy produces better results than indirect voice therapy. This same review also found that when study populations were restricted to groups of patients with specific diagnoses and assigned to well-defined voice therapy techniques, they had more success compared to studies where groups and treatments were less explicit [13]. Another review from recent times concluded that behavioral voice therapy often improves voice outcomes; however, more research into the therapeutic significance of the results is required to determine what the term effectiveness means in the context of voice therapy [12].

In this review, we analyzed the existing literature by performing an elaborate literature search to assess the efficacy of various voice therapeutic techniques among dysphonia patients. The literature search yielded a total of 17 potential studies, the details of which are illustrated and defined in Table 1 [16-32].

**Table 1:** Outcomes of included studies CPP, cepstral peak prominence; FD, functional dysphonia; FE, full vocal exercise protocol; DSI, Dysphonia Severity Index; HNR, harmonics-to-noise ratio; MCT, manual circumlaryngeal therapy; MTD, muscle tension dysphonia; NR, not reported; PE, partial vocal exercise protocol; RFF, resonant voice therapy; ref, relative fundamental frequency; VFT, vocal function exercises; VHI, Voice Handicap Index; VRT, vocal rehabilitation therapy; VT, vocal therapy; VTD, vocal tract discomfort

Author	Type of dysphonia	Voice therapy modality utilized for treatment	Outcome
Chen et al. [16]	Voice disorder	Resonant therapy	Positive effects on voice quality, vocal fold vibration, vocal fold closure, speaking flexibility, phonation effort, and functional communication
Morsomme et al. [17]	Dysfunctional dysphonia	Voice therapy	Vocal therapy is effective in treating dysfunctional dysphonia, with a high degree of satisfaction, particularly in improving vocal quality. There was a significant decrease in perceived handicap post-treatment, indicating the efficacy of vocal therapy in the long-term management of dysfunctional dysphonias.
Kleemola et al. [18]	Organic and functional	Voice therapy	A considerable proportion of patients across different severity levels experienced an improvement in voice disorder symptoms, with effect sizes indicating significant overall improvement, which persisted after therapy. Functional and organic voice disorder patients demonstrated similar improvement, although functional patients may have derived slightly greater benefit from treatment, suggesting progressive and comparable efficacy of voice treatment.
Law et al. [19]	Voice disorder	Group therapy	Group therapy as a service delivery model possesses many advantages from a psychosocial, clinical, and health resources allocation perspective.
Watts et al. [20]	MTD or phonotraumatic lesions	Stretch-and-flow voice therapy	Significant improvements in the s/z ratio, maximum phonation time, sentence CPP, and VHI through therapy were observed, with large effect sizes observed for the s/z ratio and VHI and moderate effect sizes for maximum phonation time and sentence CPP.
Ogawa et al. [21]	MTD	Humming and subsequent um-hum phonation on the computed parameters of electroglottographic	Immediate effect in adjusting the regularity of vocal fold vibration and augmenting the degree of glottal contact in MTD patients as well as nondysphonic speakers, whereas humming alone increases the degree of glottal contact in MTD patients
Steppet al. [22]	MTD	Voice therapy: RFF	Both the cycle and therapy phases significantly influence REF, with post-therapy REF measurements being significantly higher than pretherapy measurements.
Mathur et al. [23]	Hyperfunctional dysphonia	Voice therapy in teachers	Voice therapy improved voice quality.
Nguyen and Kenny [24]	MTD	Vocal function exercises	Significant changes in perturbation (HNR) in the FE group after treatment were observed, indicating a reduction in voice perturbation and improved vocal quality. The FE group showed increased size and speed of pitch change and fewer adverse effects. Both interventions showed some degree of benefit.
de Oliveira Lemos et al. [25]	MTD	Indirect and direct voice therapy	An improvement was seen in various acoustic and auditory perceptual parameters after the voice therapy intervention. A positive difference was observed for vocal jitter, which decreased from 0.46% to 0.31% post-therapy, and shimmer, which decreased from 4.58% to 3.80% post-therapy.
Tierney et al. [26]	FD	Voice therapy	Voice therapy was effective in improving voice quality in most patients with FD: 84.7% of patients achieved normal voice quality.
Sarin and Chatterjee [27]	MTD	MCT, SOVTE, and vocal hygiene program	DSI impairment levels and VHI scores showed significant improvement from the baseline to both at six weeks and three months of VRT (p < 0.001). DSI and VHI scores even showed significant improvement between six weeks and three months of therapy.
Kaneko et al. [28]	MTD	Voice therapy: flow phonation technique	Aerodynamic assessments, acoustic findings, and self-ratings demonstrated improvement following voice therapy. Stroboscopic examinations conducted before voice therapy revealed asymmetric vibration with a glottic gap, which showed improvement after voice therapy. Additionally, there was an increase in fundamental frequency (F0) post-therapy.
Başer and Denizoğlu [29]	Psychogenic dysphonia	DoctorVox voice therapy	Patients experienced significant improvements in VHI-10 values, with mean scores decreasing from 30.91 before treatment to 3.36 in the final follow-up examination. Additionally, grade, roughness, breathiness, asthenia, and strain scale scores decreased markedly post-treatment, indicating enhanced phonatory muscle function and therapy adherence facilitated by multidimensional biofeedback mechanisms.
Rangarathnam et al. [30]	MTD1	Flow phonation voice therapy	Treatment included cup-bubble blowing, gargling, and stretch and flow exercises. Voice quality was significantly improved in both treatment groups, with trends toward better voice-related quality of life. Although aerodynamic and acoustic measures did not significantly change, visual comparisons showed better laryngeal closure patterns in the flow phonation voice therapy group. These findings suggest that flow phonation exercises can be beneficial for individuals with MTD1, particularly in alleviating vocal hyperfunction and improving auditory-perceptual measures.
Guzman et al. [31]	Behavioral dysphonia	Physiologic voice therapy	The physiologic voice therapy group showed significant improvements in VHI, VoiSs, VTDS (decrease), and self-perception of resonant voice quality (increase), along with significant decreases in subglottic pressure, phonation threshold pressure, and glottal airflow across the implemented tasks. These findings suggest that physiologic voice therapy based on semi-occluded vocal tract exercises effectively enhances voice outcomes in individuals with behavioral dysphonia, particularly in physical and functional aspects, with subglottic pressure and phonation threshold pressure serving as sensitive indicators of phonatory effort reduction post-therapy.
Khoddami et al. [32]	MTD	Combined VT with other approaches	All groups undergoing VFTs, MCT, or combined VT demonstrated significant improvements in the VTD scale and DSI scores post-treatment. Additionally, a significant difference was observed between the groups (p ≤ 0.05), with the combined VT group showing the greatest improvement in the VTD severity subscale and DSI scores (η2 = 0.99 and 0.98, respectively). Notably, the interactive effect of treatment and time was significant, suggesting that combined VT yielded the most substantial improvements in MTD teachers, emphasizing the potential benefit of integrating various therapeutic approaches for the management of MTD.

The majority of the reported dysphonia cases were muscle tension dysphonia, followed by functional, organic, dysfunctional, behavioral, psychogenic, and voice disorder cases. Almost all of the included studies demonstrated the effectiveness of voice therapy in the treatment of dysphonia. Chen et al. defined that with the utilization of resonant voice therapy, positive effects on voice quality, vocal fold vibration, vocal fold closure, speaking flexibility, phonation effort, and functional communication were observed [16]. Similarly, findings from a study by Morsomme et al. demonstrated that vocal therapy is effective in treating dysfunctional dysphonia, with a high degree of satisfaction, particularly in vocal quality improvement [17]. Kleemola et al. indicated that a considerable proportion of patients across different severity levels experienced improvement in voice disorder symptoms, with effect sizes indicating significant overall improvement, which persisted after therapy [18]. Watts et al. observed that with stretch and flow voice therapy, there were significant improvements in the s/z ratio, maximum phonation time, sentence cepstral peak prominence, and Voice Handicap Index (VHI), with large effect sizes observed for the s/z ratio and VHI and moderate effect sizes for maximum phonation time and sentence cepstral peak prominence [20]. Ogawa et al. noted that with humming and subsequent um-hum phonation on the computed parameters of electroglottography, an immediate effect was seen in adjusting the regularity of vocal fold vibration and augmenting the degree of glottal contact in muscle tension dysphonia patients as well as non-dysphonic speakers, whereas humming alone increases the degree of glottal contact in muscle tension dysphonia patients [21]. Stepp et al. reported that both the cycle and therapy phase significantly influence the relative fundamental frequency, with post-therapy relative fundamental frequency measurements being significantly higher than pre-therapy measurements [22]. Mathur et al. further agreed that voice therapy improved the voice quality of patients suffering from hyperfunctional dysphonia [23]. While Nguyen and Kenny observed that with vocal function exercises, voice quality significantly improved, along with a reduction in voice perturbation [24], de Oliveira Lemos et al. also reported that with direct and indirect voice therapy, improvement in various acoustic and auditory perceptual parameters was observed [25]. A study from Tierney et al. further added that voice therapy was effective in improving voice quality in most patients [26], while findings from a study by Kaneko et al. added more evidence in this context by reporting that aerodynamic assessments, acoustic findings, and self-ratings demonstrated improvement following voice therapy [28]. Sarin and Chatterjee reported that, in addition to the vocal hygiene program, manual circumlaryngeal therapy also resulted in significant improvements [27].

Findings of a present study by Başer and Denizoğlu highlighted the efficacy of voice therapy in the management of dysphonia as patients experienced significant improvements in VHI-10 values, with mean scores decreasing from 30.91 before treatment to 3.36 in the final follow-up examination [29]. Additionally, Rangarathnam et al. suggested that flow phonation exercises can be beneficial for individuals with muscle tension dysphonia, particularly in alleviating vocal hyperfunction and improving auditory-perceptual measures [30]. Guzman et al. observed in their study that physiologic voice therapy based on semi-occluded vocal tract exercises effectively enhances voice outcomes in individuals with behavioral dysphonia, particularly in physical and functional aspects, with subglottic pressure and phonation threshold pressure serving as sensitive indicators of phonatory effort reduction post-therapy [31]. Ribeiro et al. further agreed in this context, as the authors described that voice therapy utilizing semi-occluded vocal tract exercises yielded beneficial outcomes concerning voice quality, symptoms, and musculoskeletal discomfort in females experiencing behavioral dysphonia. Rooted in the taxonomy of voice therapy, this approach appears to have fostered phonatory equilibrium, muscle release, and enhancement in vocal resilience among this demographic [33]. Another study by Khoddami et al. noted that combined voice therapy yielded the most substantial improvements in muscle tension dysphonia among teachers, emphasizing the potential benefit of integrating various therapeutic approaches for the management of muscle tension dysphonia [32].

While further analyzing the current literature, the results of a study by Mansuri et al. reported that after undergoing voice therapy, notable enhancements were noted in the acoustic attributes such as jitter, shimmer, and harmonics-to-noise ratio (p < 0.05). In terms of auditory-perceptual evaluation, there was a marked decrease in overall severity, roughness, and breathiness (p < 0.05). Thus, voice therapy appears to be efficacious in diminishing the occurrence and intensity of vocal tract discomfort in individuals with muscle tension dysphonia while also enhancing voice quality [34]. Manzoor et al. further highlighted the efficacy of this technique since the majority of the patients were satisfied with their voice therapy and deemed it easy to communicate after that [35]. Moreover, Ohlsson et al. described the long-term benefits of behavioral voice treatment, particularly in a group environment [36]. Another study by Trajano et al. also advocated that group voice therapy significantly reduced vocal discomfort and anxiety in patients with dysphonia [37]. Cohen and Garrett also agreed that voice therapy is an excellent first-line treatment for hoarseness in people with vocal fold polyps and cysts. Patients with translucent polyps, objective muscular tension dysphonia, and complete vocal fold closure on videostroboscopy may react better to voice therapy [38]. Similarly, among our included studies, Law et al. emphasized that group voice therapy as a service delivery model possesses many advantages from the psychosocial, clinical, and health resources allocation perspective [19]. Additionally, findings of a study by Cantarella et al. also reported that some dysphonia patients may benefit from group voice therapy, which can enhance perceptual, acoustic, aerodynamic, and self-evaluated characteristics. This form of treatment may help to reduce the expenditures and waiting lists connected with rehabilitative care while also increasing patients’ motivation and compliance [39]. Our review provides deep insights into the efficacy and utilization of various voice therapeutic modalities for dysphonia patients. However, our analysis of the current literature identified the dearth and scarcity of studies in this regard; moreover, the intrinsic characteristics and heterogeneity among the included studies may limit the generalizability of our results. This underscores the need for further research in this domain. Furthermore, to comprehensively characterize the outcomes of various voice therapy techniques in dysphonia treatment, particularly in comparison with traditional approaches, large-scale prospective investigations are imperative. Additionally, systematic reviews and meta-analyses can provide more conclusive findings about the effectiveness of these interventions. By synthesizing data from multiple studies, these analyses would provide robust evidence regarding the efficacy and effectiveness of different therapeutic modalities, thereby facilitating informed decision-making in clinical practice and advancing our understanding of optical therapies and treatment strategies for dysphonia.

## Conclusions

Dysphonia significantly impairs the quality of life of patients and increases the risk of depression and anxiety due to problems in communication; therefore, early diagnosis and prompt management are vital for the well-being of patients. Voice therapy is beneficial for the treatment of dysphonia, which does not necessitate surgical intervention and is proven to exhibit improvement and optimal outcomes, thus improving the quality of life of these patients. However, the evidence available in the literature is limited, which necessitates further research to address and analyze the efficacy of various voice therapeutic techniques among dysphonia patients, specifically so more evidence-based findings from recent times are available to guide clinical practice.
